# Multilingual Competence Influences Answering Strategies in Italian–German Speakers

**DOI:** 10.3389/fpsyg.2018.01971

**Published:** 2018-10-31

**Authors:** Irene Caloi, Adriana Belletti, Cecilia Poletto

**Affiliations:** ^1^Institut für Romanische Sprachen und Literaturen, Goethe-Universität Frankfurt am Main, Frankfurt am Main, Germany; ^2^Dipartimento di Scienze Sociali, Politiche e Cognitive, Università di Siena, Siena, Italy; ^3^Départment de Linguistique, Faculté des Lettres, Université de Genève, Geneva, Switzerland; ^4^Dipartimento di Studi Linguistici e Letterari, Università degli Studi di Padova, Padova, Italy

**Keywords:** heritage language, L1 attrition, new information subjects, interfaces, optionality

## Abstract

The present study aims at analyzing the role of nativeness, the amount of input in L1 acquisition and the multilingual competence in the performance of Italian–German bilingual speakers. We compare novel data from the performance of adult L2 learners (L1: Italian; late L2: German) and that of heritage speakers (heritage language: Italian; majority language: German) to previous data from monolingual speakers of Italian. The comparison deals with the produced word order at the syntax-discourse interface in sentences containing New Information Subjects in answers to questions that prompt the identification of the clausal subject. Overall, adult L2 speakers and heritage speakers perform alike but crucially differently from Italian monolinguals. These data reveal that multilingual proficiency determines an increased variety in the adopted answering strategies; in particular, the German-like strategy is active in Italian. Nativeness alone is thus no guarantee for a homogeneous performance across groups, nor do we find similar patterns of performance in speakers who grew up as monolinguals. Data also show heritage speakers’ sensitivity to verb classes, with answering strategies varying in accordance with the verb argument structure. Participants’ productions reveal an interesting relation in sentences with transitive verbs between subject position (pre-/postverbal) and object form (lexical DP/clitic pronoun).

## Introduction

This study addresses the issues of the role of nativeness, the amount of input in L1 acquisition and the multilingual competence in the performance of Italian–German bilingual speakers. We compare novel data from the performance of adult L2 speakers possibly undergoing attrition (AL2S; L1: Italian; late L2: German) and that of heritage speakers (HSs; heritage language: Italian; majority language: German) to previous data from monolingual speakers of Italian (MonoL1; Belletti and Leonini, 2004).

For the purpose of the present study, we rely on a concept of nativeness that corresponds to the exposure to the target language since birth in the familial environment, independently of the proficiency level ultimately attained later. As for the amount of input in L1 acquisition, we refer to the different linguistic settings in which the L1 is acquired. In a multilingual setting children receive an input in the heritage language that is not as rich and differentiated as that of monolingual children. The third factor we address, i.e., multilingualism, takes into consideration the linguistic competence of our participants, who are advanced speakers of both Italian and German at the time of testing, independently of age of acquisition (bilingual child acquisition or adult L2 acquisition of German).

The three factors (although termed slightly differently) have been previously singled out by Montrul (2016, p. 17) as important variables in defining speakers’ linguistic dominance. Along the lines of Kupisch and Weijer (2016, a.o.), we can refer to dominance as the strongest language in the speakers’ competence. According to Montrul’s model, proficiency is only one aspect of dominance, which usually correlates with other biographical and input variables (such as age of acquisition, place of birth, amount of input, type of context, etc.). Here, we will address the relationship between these variables and the attained proficiency. However, given that proficiency can vary depending on the linguistic level of analysis, we specify at the outset that we will focus on one specific phenomenon, rather than addressing a general linguistic assessment. The idea is to gain a better understanding of the role of single variables through the study of their reflex on a single linguistic phenomenon. This should ultimately contribute to highlight how dominance may be (re)set throughout the lifespan.

Our analysis addresses the produced VS and SV word orders determined at the syntax-discourse interface in sentences that are the reflex of a specific discourse content: the realization of New Information Subjects (NISs) in answers to questions that prompt the identification of the clausal subject.

We present the phenomenon referred to as *answering strategies* in Section “Answering Strategies.” In Section “Subjects at the Interfaces: Previous Results From Multilingual Speakers” we discuss previous results from the performance of multilingual speakers in phenomena related to the syntax and interpretation of subjects at the interface with discourse in question–answer contexts. On this basis we will formulate our research questions in Section “Research Questions.” In Section “Materials and Methods” we present the methods we used to collect the results presented in Section “Results.” Section “Discussion” is dedicated to the discussion of the results. Section “Conclusion” gives the conclusions.

## Answering Strategies

In the present study, we consider a linguistic phenomenon that manifests itself at the interface between syntax and discourse, and we look at how a specific interpretive content is conveyed in the syntactic structure. Specifically, we are interested in NISs, which result from question–answer pairs aiming at identifying the clausal subject. The specific linguistic phenomenon is triggered by questions of the following kind:

(1) Chi ha vinto il premio?
    who AUX win.PP the prize
    ‘Who won the prize?’

As exemplified, the question bears on the subject, which must be identified in the answer as the Focus of New Information, while the predicate and the object are presupposed in the given conversational context. As discussed in Belletti (2007) and in Belletti and Leonini (2004), languages differ in the way they answer questions that trigger NISs, i.e., they resort to different syntactic structures and different word orders to convey the intended meaning. Following the references quoted, we will refer to those selected structures as *answering strategies*.

Three main answering strategies have been identified in the languages investigated: verb–subject (VS) order with a postverbal subject; subject clefts, and subject–verb (SV) order with the preverbal subject bearing a characteristic prosodic prominence. The strategy preferably adopted in Italian exploits the VS order with a postverbal subject as in (2)A (Belletti, 2001, 2004, a.o.):

(2) Q: *Chi ha vinto il premio?*
        who AUX win.PP the prize?
    A: *L’ ha vinto Maria*
        obj.CL AUX win.PP M.
    Q: ‘Who won the prize?’ A: ‘Maria won it’

In the same pragmatic context, French native speakers tend to produce subject clefts or reduced clefts (Belletti, 2007):

(3) Q: *Qui a gagné le prix?*
        who AUX win.PP the prize?
    A: *C’est Marie (qui l’ a gagné)*
        CL be M. who obj.CL AUX win.PP
    Q: ‘Who won the prize?’ A: ‘It is Marie (who won it)’

In contrast, other languages like English and German tend to focalize the NIS in the preverbal position associating it to a marked prosodic prominence, as exemplified in the German example in (4):

(4) Q: *Wer hat den Preis gewonnen?*
        who AUX the prize win.PP
    A: *MaRIE hat ihn gewonnen*
        M. AUX OBJ. ProN win.PP
    Q: ‘Who won the prize?’ A: ‘Maria won it’

The three examples show how the same discourse function, i.e., the realization of NISs in answers to questions that aim at identifying the clausal subject, is carried out in different languages through strategies that differ in their syntactic structure and in their prosodic pattern. We assume with Belletti (2001, 2004, and subsequent work) the cartographic analysis according to which the low vP-peripheral area of the clause (TP) contains a discourse related Focus position dedicated to the New Information Focus interpretation and Topic positions along similar lines as the clause external Left periphery (Rizzi, 1997; Benincà and Poletto, 2004; Cruschina, 2009, 2012, and much subsequent work). As for the Italian example in (2)A, the structure is assumed to be obtained through the activation of the Focus position in the clause-internal vP-periphery, dedicated to host new information constituents, hence the NISs as well. According to this analysis, the low vP-peripheral position hosting the NIS is lower than the position targeted by the verb in its (head) movement within the TP. Thus, the VS order is obtained with V moving over the low NIS along the lines schematically illustrated in (5):


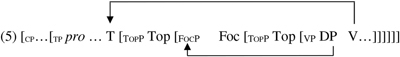


In a null subject language like Italian, a silent referential *pro* is present in the high preverbal subject position, satisfying the relevant formal requirements (i.e., EPP, Chomsky, 1995; Cardinaletti, 2004; Rizzi and Shlonsky, 2007).

The structure of answering strategies with a postverbal subject ultimately relies on a number of syntactic mechanisms: the activation of the clause-internal low FocusP position for hosting the NIS, movement of V to T^1^ and presence of *pro* in the preverbal subject position. It follows that the strategy cannot be exploited if the language is not a null subject language^2^. However, if some other way is available to satisfy the non-null subject property of the language, i.e., if the preverbal high subject position may be filled otherwise, for instance by an expletive subject pronoun, then the NIS can be left in the specifier of the low Focus position also in a non-null subject language, without violating any grammatical constraint. French cleft sentences of the type presented in (3) above illustrate one such case of how a non-null subject language can exploit the low vP-peripheral new information Focus position to express a NIS, essentially implementing a postverbal subject in disguise (Belletti, 2005, 2009, 2015; see also Hamann and Tuller, 2014, for an overview on French cleft and presentational constructions)^3^. In a nutshell (the relevant steps of) the derivation runs as follows. Let us assume that the core structure of clefts is built by a matrix clause containing the copula with its vP-peripheral discourse related projections including the New Information Focus one. The copula in turn takes as its complement a small clause (reduced) CP. The subject leaves its original position within the small clause CP and moves to the specifier of the low FocusP position in the vP-periphery of the copula in the matrix clause. Further movement of the copula to its functional T position results in the familiar VS word order. Finally, the high preverbal subject position is filled by the quasi-expletive pronoun *ce*. The basic features of the French subject-cleft answers are illustrated in (6):

(6) [_TP_ *Ce est*_i_ [_FOCP_ *Marie*_j_ [_V P_ <___i_> [_SM_ [_PREDP_ [_FinP_ *qui* [<___j_> *a gagné le prix*]

Thus, Italian and French answering strategies to questions that trigger NISs, i.e., VS with a postverbal subject and subject clefts, both require the activation of the very same New Information Focus position in the vP-periphery. Cross-linguistic data ultimately offer robust evidence for the presence of such a position and its activation under the described discourse pragmatic conditions.

As mentioned above, at least one further strategy is attested cross-linguistically and that does not imply the activation of the low vP-peripheral Focus position, namely the focalization of the subject in preverbal position through prosodic prominence. The strategy is attested in Germanic languages, such as, e.g., English and German, but also in Romance languages such as Brazilian Portuguese (Dal Pozzo and Guesser, 2010) and, as recently discussed, in South American varieties of Spanish (Gabriel, 2010; Hoot, 2012; Leal et al., 2017), as well as in Bulgarian (Genevska-Hanke, 2017; see footnote 2). The strategy consists in having the subject in preverbal position, yielding the SV linear order, and in attributing a characteristic prosodic prominence to the preverbal NIS.

In conclusion, given this brief summary, it clearly emerges that the shaping of an answering strategy is an articulated task, crucially involving both syntactic computations and their relation with the prosodic and interpretive interfaces^4^.

## Subjects at the Interfaces: Previous Results From Multilingual Speakers

Previous studies reported that multilingual speakers show optionality and non-target-like outputs for phenomena at the interface between syntax and pragmatics, such as, e.g., Topic shifts and NISs.

Sorace (2005, 2011) proposed the so-called Interface Hypothesis to provide a possible explanation for such results: phenomena that imply the integration of information from different cognitive systems may be more prone to instability in multilingual speakers [‘unstable domains’ in Sorace’s (2011, p. 3) terms]. For instance, whereas Italian monolingual speakers agree in interpreting overt subject pronouns of subordinate clauses as Topic shift with respect to the main clause, thus selecting a referent different from the subject in the preceding matrix clause, English–Italian bilingual children (Sorace et al., 2009), attrited L1 speakers (Tsimpli et al., 2004), and advanced L2 learners of Italian (Belletti et al., 2007) show higher acceptance of coreference of the overt subject pronoun with the subject of the previous sentence, thus disregarding Topic shift ^5^. Furthermore, the different types of bilingual/L2 speakers investigated may have access to a different possible grammatical analysis of the overt subject pronoun as a weak pronoun (in the sense of Cardinaletti and Starke, 1999; Cardinaletti, 2004), as the weak overt pronouns of their other language (e.g., she/he) are the equivalent of the Italian non-overt *pro*. Since the topic-continuity interpretation is available in their other language in the presence of an overt weak subject pronoun, bilingual/L2 speakers may overextend this interpretation also to overt Italian pronouns; however, in the same context monolinguals tend to prefer the weakest subject, i.e., the null variant, while overt subject pronouns tend to be interpreted as Topic-shift (Belletti et al., 2007, p. 672).

Given that answering strategies concern the production of subjects which express new information Focus at the discourse level, they also qualify as a potentially unstable domain in the linguistic performance of multilingual speakers. Belletti and Leonini (2004; see also Leonini and Belletti, 2004) tested which answering strategies adult learners of Italian produce when prompted to realize NISs. Their experimental group was not homogenous from the point of view of the participants’ L1s and this clearly had a reflex on their different outputs, thus offering a straightforward interpretation of the results. Sixteen participants were German native speakers, who produced the target-like VS structure only in the 27% of the experimental items; while in the 68% of their answers they produced SV structures, with the focalized subject in the preverbal position. Three L2 learners with L1 French frequently produced answers in Italian with French-like clefts (69%) in order to express NISs^6^, in accordance to what has been described in Section “Answering Strategies” as the prototypical answering strategy in French. Thus, L2 speakers recognized the appropriate discourse context for the realization of NISs and reacted by activating an answering strategy which was not the one most prominent in the L2. Finally, Belletti and Leonini (2004) enrolled seven L2 learners with different L1s (e.g., Greek, Albanian, Polish); the group was very successful at producing target-like outputs of the VS kind (91%) across all verb types (range 87–93%). All participants in the third group were native speakers of null subject languages, which should explain their success at producing the target VS answering strategy^7^, in contrast with the German and French groups. As discussed in Section “Answering Strategies,” the possibility to focalize the subject in the postverbal position crucially relies on the availability of a silent *pro* in the preverbal subject position, i.e., the null subject property, a necessary (although not sufficient, see footnote 2) condition for VS. The low production of VS structures shown by the French and German groups might be interpreted as a difficulty for learners to take into account all syntactic properties of *pro*, which finally results in the unsuccessful resetting of the null subject parameter. However, that this cannot be the case is indicated by the fact that null subjects in Italian are largely available in both groups. Hence the conclusion must be that access to VS under the appropriate discourse conditions and availability of null subjects do not go together in L2 acquisition (see footnote 2 and the discussion in Belletti and Leonini, 2004). To sum up, results from this first study show that the L1 answering strategy remains active in adult L2 learns: when the production of NISs is elicited in L2 Italian, German speakers mainly produce SV answers, French speakers generally produce clefts, and only native speakers of null subject languages are successful in producing the target VS answer.

Postverbal subjects are hardly produced also by advanced learners who qualify as near-native speakers of Italian and have either British or American English as their L1 (Belletti et al., 2007). Results reveal that, despite their advanced acquisition of Italian, their use of the target-like VS strategy is still rather limited. Participants tend to produce SV structures with preverbal subjects focalized through prosodic prominence, in line with the dominant strategy in their L1^8^. Moreover, data from further independent tests run in the quoted study show that participants can use null subjects in an appropriate way^9^. This confirms the conclusion already drawn for non-advanced speakers of Italian reviewed above: most L2 speakers of Italian (with English-, German-, and French L1) show a dissociation between the use of null subjects and the production of postverbal subjects, with the latter not showing any significant development in time when the L1 is a non-null subject language. Overall, data from the two reviewed studies show a persisting difficulty in the production of target-like VS structures, with a parallel persisting activation of the prominent strategy of the native language (i.e., SV for English and German speakers).

In conclusion, the aspects of the background literature on the mastering of properties of subjects in Italian by multilingual speakers, relevant for the present study can be summarized as follows. Firstly, native speakers of Italian might show signs of attrition in this domain. The phenomenon, reported in Tsimpli et al. (2004) mentioned at the outset of this section, concerns Italian native speakers who qualify as near-native speakers of English and show altered interpretation of overt subjects. Whereas monolingual speakers of Italian interpret overt pronominal subjects of subordinate clauses as instantiations of Topic-shift with respect to the matrix clause subject, L2 speakers show a higher acceptance of coreference of the two subjects. Thus, attrition manifests itself in those speakers in the form of a broader acceptance of overt pronouns. As this work showed changes and attrition in the interpretation of subjects with respect to their overt/non-overt pronominal realization, this further encourages us to investigate whether another discourse-related property, i.e., the pre-/postverbal position of the overt subject might similarly undergo attrition.

Despite correct use of the null subject property, L2 learners of Italian show persisting difficulties at achieving a target-like use of the related property yielding the order VS in answers containing a NIS even at advanced stages of acquisition. The fact that target answers are correctly produced in the second language only by those speakers whose L1 is a null-subject language suggests that the successful acquisition of the relevant answering strategy might be dependent on an early setting of the parameter in child acquisition. In this line of reasoning, we speculate on the idea that the native answering strategies cannot be easily inhibited in L2, especially in the case in which these lead to the production of grammatical sentences in the target language (although infelicitous in the given context as, e.g., use of SV instead of VS in Italian).

Results from the two groups, i.e., attrited native speakers and L2 speakers, seem to point to two different hypotheses for the mechanism that shapes answering strategies. On the one hand, L1 attrition within the domain of pronominal subject interpretation shows that this is actually an unstable domain, whose system can be influenced by advanced L2 acquisition. In this vein, we can hypothesize that multilingualism shapes answering strategies in forms that depend on the properties of the languages involved. On the other hand, the observation of L2 learners suggests the hypothesis that answering strategies are crucially shaped in childhood, and L2 learners experience difficulties in inhibiting the native strategy when this offers grammatical options in the L2 (as it is the case for subject clefts and SV structures in Italian). We can therefore hypothesize that answering strategies are in place from very early ages and eventually keep stable despite the presence of competing L2 grammatical options, thus turning nativeness and amount of input in L1 acquisition into the crucial factors for shaping answering strategies. If this hypothesis is correct, we should find an Italian native-like performance in our multilingual speakers. We take into consideration both hypotheses in what follows by analyzing the role of multilingualism, nativeness and amount of input in L1 acquisition in answering strategies.

## Research Questions

In the previous section, we sketched out two plausible routes for the shaping of answering strategies in multilingual speakers. In order to enlarge the body of data at our disposal and to analyze the two hypotheses, we run a test on the production of answering strategies in two groups of multilingual speakers who share the same languages. All our participants are Italian–German speakers; however, for one group of speakers, Italian is the native language and German is the L2; for the second group of participants, Italian is the heritage language and German is the majority language. We refer to the first group as adult L2 speakers (AL2S), and to the second group as HSs. To the best of our knowledge, these two categories with the Italian–German combination have never been tested before for the production of answering strategies. Together with data from previous studies on L2 acquisition (Belletti and Leonini, 2004; Leonini and Belletti, 2004) and on near-native speakers of Italian (Belletti et al., 2007), we will complete the picture of how answering strategies are computed by Italian–German multilingual speakers. In particular, by choosing participants who speak Italian and German in different settings and with different acquisition histories (adult L2 acquisition of German, or Italian as heritage language), we want to investigate how different factors contribute in shaping answering strategies. In particular, the factors that we take into consideration are nativeness, amount of input in childhood, and multilingualism.

As pointed out by Kupisch and Rothman (2016, a.o.), the opposition between HSs and native speakers is not correct from the theoretical point of view; in fact, HSs are also native speakers of the heritage language. In other words, they cannot be opposed to native speakers, because they are native speakers of the heritage language themselves. From this point of view, HS, AL2S and monolingual speakers do not differ, because they all started acquiring Italian from birth in the familial environment, such that they can all be considered native speakers of Italian. If being a native speaker is the crucial factor in shaping answering strategies, we should find homogeneous performances across speakers’ profiles. However, one important difference among the groups might be the amount of input received during L1 acquisition. Monolingual speakers and AL2S all grew up only with one language; therefore, we can assume that they all received a comparable amount of input in the critical period. In contrast, HSs grew up in a community characterized by a majority language other than the heritage language. Hence, the input they received in the heritage language is different from that received from the two groups who grew up in a monolingual setting in Italy, both from the quantitative and the qualitative point of view. Although this is subject to extreme individual variation, HSs have on an average a more limited access to the heritage language because the majority language usually covers some relevant communicative functions (e.g., education, interaction with peers in public spaces, TV shows, etc.). As for the linguistic phenomenon at stake, HS are exposed to both answering strategies (VS and SV answers) in the input through the two languages. By comparing the three groups of speakers, i.e., monolingual speakers of Italian and adult L2 speakers (of German, L1 Italian) on one hand, and HSs (of Italian, German the majority language) on the other hand, we aim at verifying the role played by the input during language acquisition in shaping answering strategies.

Moreover, subtle differences between AL2S and HS might emerge in the two groups as an effect of different syntactic conditions. As briefly mentioned in Section “Subjects at the Interfaces: Previous Results From Multilingual Speakers,” previous studies already reported that the argument structure of the verb in use could have an influence on the adopted answering strategy (see Belletti and Leonini, 2004; Belletti et al., 2007). The analysis of the collected data will take into consideration the verb class as a relevant factor in order to draw a comparison between the experimental groups. Differences among verb classes might reveal further interesting aspects of the performance of multilingual speakers.

For the same reason, we want to add one further research question, which concerns the realization of objects in transitive structures. Although the study focuses on the production of NISs, structures containing transitive verbs also offer the opportunity to observe how the internal argument is produced in the specific discourse context. In fact, the object is usually given in the question in use for triggering the answering strategy with NISs, such that the element is a Topic and should be realized as a pronoun in the answer, specifically as a clitic pronoun in a language with clitics like Italian. We will observe which strategy multilingual speakers put in use to convey Topic-like objects and their interaction with NISs.

To sum up, in the present study we intend to address the following research questions:

(i) Do multilingual speakers produce different answering strategies with respect to monolingual speakers of Italian?
(ii) If so, does the type of acquisition setting (monolingual/bilingual) play any role in shaping answering strategies? To what extent do the strategies for NISs realization differ in the two groups?
(iii) Does the verb class affect the type of answering strategy activated by participants?
(iv) How are objects realized when the answer contains a transitive verb?

With respect to questions (i) and (ii), we can make the following speculations: if nativeness is the decisive factor in shaping answering strategies, we expect AL2S and HS to perform similarly to MonoL1 speakers. Alternatively, it could also be argued that the decisive factor is the amount of input received during L1 acquisition; if that is the case, we expect AL2S and MonoL1 to perform similarly, but not HS. Finally, if multilingualism, and the subsequent presence of both conflicting strategies in the input through the two languages, is the decisive factor, we should find a third pattern of performance: HS and AL2S performances should pattern alike, but crucially differ from that of MonoL1. The pattern of performance across the three experimental groups (MonoL1, AL2S, and HS) should reveal which factor among nativeness, amount of input in L1 acquisition, and multilingualism plays the bigger role in shaping answering strategies. Moreover, results could shed light on the kind of input received by HS in the multilingual environment they grew up in, since native speakers with an advanced command of the L2 (and therefore potential attrition of the L1) ultimately represent the privileged source of input for HS.

## Materials and Methods

### Participants

The following groups of speakers took part in the present study: 22 adult heritage speakers of Italian (HS) with German as the majority language, and 20 adult L2 speakers (AL2S), who are native speakers of Italian and acquired German as L2 after childhood. Their results will also be compared to those of a group of monolingual speakers of Italian (MonoL1), whose data are reported from the original study presented in Belletti and Leonini (2004).

Heritage speakers grew up in Germany and were exposed to Italian since birth by either one or both parents, who were native speakers of Italian^10^ and used the language in the daily interaction with the child (see Rothman, 2009). At the time of testing all HS reported to use Italian as the family language together with German. Except for two speakers whose parents had moved away from Italy during childhood, all other HS participants were second generation children of parents who left Italy in their early adulthood. HS participants were educated in the majority language, but 20 of them (out of 22) also took formal courses of Italian at school and/or at the university. Based on these data, we assume that HS have received a reduced amount of input in the heritage language with respect to monolinguals^11^. At the time of testing, all participants were enrolled as undergraduate students at the Goethe University of Frankfurt. No minimum level of proficiency in Italian was required to take part in the study. However, in choosing participants for this study we particularly took into consideration two factors: formal education and contact with the family of origin. First, in line with the studies discussed in Kupisch and Rothman (2016), we assume that formal education in the heritage language and literacy can allow for higher proficiency and closer to monolingual-like performance. Second, the young age of the participants translates into closer and on-going relationships to their families of origin, which plays a relevant role in heritage language maintenance. As previously pointed out by O’Grady et al. (2011) and by Polinsky (2011), heritage language attrition can take place over the life-span as an effect of reduced contacts to the family and the community of origin, i.e., reduced use of the heritage language in everyday life. We aimed at recruiting high performing participants by choosing young adults who are (or have been) engaged in formal courses of Italian on a regular basis and who maintain a tight contact with the family of origin in which Italian is spoken. We expect the enrolled participants to have benefitted from their exposure to multiple diversified speakers in a number of varieties and registers during their education and from the contacts with the family of origin and, possibly, with the Italian community. Although the two factors will not be analyzed as experimental factors in our analysis, we point out that, based on previous results from the literature (O’Grady et al., 2011; Polinsky, 2011, a.o.; Kupisch and Rothman, 2016), they were included in the guidelines for the recruitment of high-performing participants.

Adult L2 speakers (AL2S) were native speakers of Italian, who grew up in Italy as monolingual speakers of Italian, moved to Germany as young adults (most AL2S moved to Germany during University years), and learnt German as L2. In most cases, relocation to Germany was preceded by some formal courses of German as L2 taken at Italian schools or universities. Participants from this group all reported using German on a daily basis both at work and at home, although in variable amounts. The same holds true for Italian: they all reported to use Italian daily, in familial interactions and/or in a variety of entertaining and social activities (e.g., watching/reading the news, films, books, social media interaction, etc.). Although to the writers’ knowledge there is no acknowledged minimum amount of exposure time to the L2 in order to allow for the onset of attrition effects, we nonetheless set the requirement of a minimum of 4 years of continuative stay in Germany with the described systematic use of German for AL2S participants to enroll in the study.

The characteristics of HS and AL2S participants are summarized in Table 1, together with the information on the monolingual speakers, whose data are reported from Belletti and Leonini (2004).

**Table 1:** Participants’ characteristics (data for MonoL1 are reported from Belletti and Leonini, 2004).

Participants	N°	Age range Mean (SD)	Years in Germany (SD)	Cloze-t IT (SD)	Cloze-t GE (SD)
AL2S	20	26–60 41;1 (8;3)	13;7 (5;1)	85.5% (2)	69% (5)
HS	22	21–38 25;6 (5)	25;6 (5)	56.4% (3)	77% (1)
MonoL1	10	20–33	–	–	–

As reported in Table 1, AL2S were older than HS on average: this is intrinsically due to the characteristics of the participants we decided to enroll. On the one hand, AL2S learnt German as L2 after childhood, moved to Germany as young adults, and certainly needed some years in order to achieve a good competence of German. On the other hand, HS are young undergraduate students, who still live with the family of origin in most cases. This explains why it would have not been possible to match the two participant groups for age.

In the fourth column, we report data on the amount of years spent in Germany by participants in the two groups. On average, AL2S speakers had spent 13;7 years in Germany at the time of testing (range: 4–28;3 years). As for HS, the data roughly corresponds to their age, as they spent all their lives in Germany, except for medium-term periods of stay in Italy (less than 1 year^12^).

Both groups underwent two cloze tests, one for Italian and one for German: both versions of the test consisted in a text from a newspaper article^13^, from which several functional and lexical words were erased. Participants were requested to fill in the gaps. Despite the absence of at ceiling performances, AL2S completed the Italian version of the test better than the German one, whereas HS showed the opposite pattern, with better performance in the German than in the Italian test. Results from the two groups differ in the Italian test (interval of accuracy in AL2S = 0.80–0.90; and in HS = 0.49–0.63), but not in the German one (interval of accuracy in AL2S = 0.57–0.80; and in HS = 0.74–0.81). We take the results of AL2S on the German test as a proof of their good command of the L2.

As to MonoL1 participants, Belletti and Leonini (2004) tested 10 native speakers of Italian, who came from different Italian regions. Their age ranged between 20 and 33 years old. No cloze test was performed in the original study as their command of Italian was evident.

### Materials

We collected data on answering strategies through the elicited production task first presented in Belletti and Leonini (2004).

Participants watched 22 short videos and listened to 40 experimental questions, which triggered answers with NISs. Videos depicted characters involved in daily activities and ended with one of the actors asking a question on the identification of the subject. One or two further questions were also audio played at the end of each video concerning the event represented in the scene to serve as distractors. Participants were instructed to produce oral answers expressing the verb (thus allowing for the observation of the subject position); they were also explicitly encouraged to answer the questions in the way that sounded the most natural to them.

Experimental questions are distributed across four conditions, i.e., 20 finite sentences containing a transitive verb (7), 4 sentences with an unaccusative verb (8), 10 sentences with an unergative verb (9), and 6 sentences featuring existential structures with the Italian copula (10).

(7) Chi ha aperto la finestra?
    who AUX open.PP the window
    ‘Who opened the window?’

(8) Chi è arrivato?
    who AUX arrive.PP
    ‘Who arrived?’

(9) Chi ha urlato?
    who AUX scream.PP
    ‘Who screamed?’

(10) Cosa c’ è sopra il tavolo?
    what CL AUX on the table
    ‘What is on the table?’

The test material also included 19 fillers in the form of questions that concerned the video content but did not trigger answers with NISs. Experimental questions and fillers were randomized throughout the task.

### Procedure and Coding

Participants were tested individually and the test took approximately 12–15 minutes. Their outputs in the elicited production test were recorded with an aLLreLi (ALLCP0033_Q9G) digital voice recorder and later transcribed and coded by a researcher. Data from the cloze tests described above were coded by two students: a native Italian speaker coded the data from the Italian cloze test, while a native German speaker coded the data from the German cloze test. All results were cross-checked by a second researcher.

Since the goal of our study is the observation of the position of NISs, only answers containing (at least) the subject and the verb were relevant for our analysis. Therefore, all subject-only answers were discarded (11). In addition, non-relevant answers were excluded from our analysis; i.e., answers that did not contain the required Subject of New Information (12–13).

(11) La ragazza
    the girl
    ‘The girl’

(12) Non ho visto
    not AUX see.PP
    ‘I didn’t see it’

(13) Non lo so
    not CL know
    ‘I don’t know’

Only main clauses containing the subject and the verb were analyzed and classified under either one of the three following categories, depending on the word order and the syntactic structure: VS answers (14), SV answers (15), and Other answers (16–17). The label Other was used for any grammatical sentence, whose syntactic structure did not correspond to the SV or VS word order. It turned out that the majority of them were (reduced) clefts (16) or passive structures (17).

(14) L’ ha aperta la ragazza
    obj.CL AUX open.PP the girl
    ‘The girl opened it’

(15) La ragazza l’ ha aperta
    the girl Obj.CL AUX open.PP
    ‘The girl opened it’

(16) Era la ragazza
    was the girl
    ‘It was the girl’

(17) La finestra è stata aperta dalla ragazza
    the window AUX.be.pass open.PP by-the girl
    ‘The window was open by the girl’

Answers with transitive verbs were also further analyzed depending on whether the clausal object was produced as a clitic pronoun [see example (14–15) above] or as a lexical DP, as in (18):

(18) La ragazza ha aperto la finestra
    the girl AUX open.PP the window
    ‘The girl opened the window’

One further aspect we took into consideration with transitive verbs is the position of the object with respect to the verb and the subject (e.g., SVO or VOS); the issue will be addressed in details in the “Results” section.

## Results

Outputs from participants in the AL2S and HS groups were transformed into percentages according to the answering strategy in use (VS/SV/Other) in order to allow for a comparison with results from the MonoL1 group (reported from Belletti and Leonini, 2004). Table 2 offers a descriptive overview of the results from the three groups.

**Table 2:** Production of VS/SV/Other answers by MonoL1/AL2S/HS (in percentages, SD, median).

	MonoL1	AL2S	HS
VS	98	61.59	50.52
SD	2.87	26.31	27.95
Median	98.5	66.66	51.25
SV	1.0	26.03	44.75
SD	2.16	27.91	28.78
Median	0	11.36	45.97
Other	1.0	12.38	4.73
SD	1.54	19.88	8.81
Median	0	3.03	0

The data analysis was carried out using SPSS Statistics Version 17.0. Pairwise comparisons were run in order to analyze the outputs of the three experimental groups. Data revealed that both AL2S and HS perform differently from MonoL1 in the production of VS and SV answers. First, we compared AL2S against MonoL1. The Mann–Whitney tests indicated that the number of VS answers is higher for MonoL1 speakers (*Mdn* = 98.5) than for AL2S speakers (*Mdn* = 66.66), *U* = 3.00; *p* < 0.001; *r* = 0.78. In turn, more SV answers were produced by AL2S (*Mdn* = 11.36) than by MonoL1 (*Mdn* = 0), Mann–Whitney *U* = 17.50, *p* < 0.001, *r* = 0.67. No significant difference was found in the production of Other answers between MonoL1 (*Mdn* = 0) and AL2S (*Mdn* = 3.03), Mann–Whitney *U* = 67.00, *p* = 0.155, *r* = 0.28. Second, we compared HS against MonoL1. The Mann–Whitney tests indicated that the number of VS answers is higher in MonoL1 (*Mdn* = 98.5) than in HS (*Mdn* = 51.25), *U* = 5.000, *p* < 0.001, *r* = 0.75. The SV answering strategy was more frequent in HS participants (*Mdn* = 45.97) than in MonoL1 speakers (*Mdn* = 0), Mann-Whitney *U* = 6.500, *p* < 0.001, *r* = 0.74. The two groups did not differ in the production of Other answers (MonoL1 *Mdn* = 0, HS *Mdn* = 0, Mann–Whitney *U* = 92.000, *r* = 0.14). Based on these results, we conclude that both AL2S and HS perform differently from MonoL1 when producing elicited answering strategies with NISs.

We also compared AL2S and HS and found that the latter produce more SV (HS *Mdn* = 45.97) than the former (AL2S *Mdn* = 11.36), Mann-Whitney *U* = 137.00, *p* = 0.037, *r* = 0.32. As for the production of VS and Other answers, no significant difference was found between the two groups of multilingual speakers.

Table 3 shows how participants’ outputs distribute across answering strategies with respect to the kind of verb included in the question–answer pairs, i.e., transitive verbs, unergative verbs, unaccusative verbs, and existential structures.

**Table 3:** Production of VS/SV/other answers by MonoL1/AL2S/HS in sentences with transitive, unergative, unaccusative verbs, and existential structures (in percentages).

	MonoL1			AL2S			HS		
	VS	SV	Other	VS	SV	Other	VS	SV	Other
Transitive	97.00	2.00	1.00	50.00	30.34	19.66	36.82	55.81	7.37
Unergative	99.00	1.00	–	66.49	26.63	6.88	49.77	46.37	3.86
Unaccusative	95.00	5.00	–	70.00	30.00	–	60.23	39.77	–
Existential	96.00	–	4.00	99.00	1.00	–	91.51	5.31	3.18

Since MonoL1 speakers performed very consistently (see Table 3) across syntactic conditions (VS range 95–99%; SV range 0–5%, Other range 0–4%), we are going to set this group apart for a moment in order to focus our analysis on multilingual speakers (AL2S and HS).

Mann–Whitney tests revealed that the use of SV answers significantly differs between AL2S and HS in sentences with transitive verbs and with unergative verbs. As for transitive verbs, the Mann–Whitney test showed that the production of SV answers is greater in HS participants (*Mdn* = 60.0) than in AL2S participants (*Mdn* = 11.1), *U* = 129.00, *p* = 0.022, *r* = 0.35. HS participants also produce more SV answers than AL2S speakers in sentences with unergative verbs (HS *Mdn* = 47.2, AL2S *Mdn* = 12.5, Mann–Whitney *U* = 349.50, *p* = 0.041, *r* = 0.31). Finally, no significant difference between the two groups is attested when the elicited answers contain an unaccusative verb or an existential structure. The two groups (AL2S and HS) produce comparable numbers of VS and Other answers across all verb types (no significant difference revealed by Mann–Whitney tests).

We also wanted to look at data from a different perspective in order to analyze the distribution of VS and SV answers across conditions, and to check for relations between verb types and sentence structures. Our intention was to verify whether the argument structure of the predicate in use in the question–answer pairs plays a role in determining how NISs are produced, with respect to the overall activated answering strategy and in particular to the subject position.

It is evident from the data presented above that a strong relation holds for at least one condition, namely the one with the Italian copula for existential structures: in this condition, participants from both the AL2S group and the HS group are very consistent in replicating the VS word order, with the NIS following the copula (AL2S *mean* = 99.00%; *SD* = 4.35, *Mdn* = 100; HS *mean* = 91.51%; *SD* = 22.9; *Mdn* = 100). The pattern is very robust and alternative strategies are attested very infrequently. As for the remaining conditions, i.e., transitives, unergatives, and unaccusatives, no such straightforward result is observable.

Kruskal–Wallis tests did not reveal any significant difference within AL2S speakers in the production of VS and SV answers with transitive, unergative and unaccusative verbs. Based on this observation, we conclude that verb type does not play a role in determining which answering strategy is adopted by AL2S (not counting existential structures).

In contrast, data from the HS group reveal one interesting property: although the number of SV answers is stable across conditions (no significant difference revealed by Kruskal–Wallis tests), that of VS answers is not (see Table 4). From the descriptive point of view, the number of VS structures is at its lowest alongside transitive verbs (36.82%), it increases with unergatives (49.77%) and becomes significantly higher with unaccusative verbs (60.23%). The Kruskal–Wallis test revealed that there was a difference between the number of VS answers produced with different verb types not quite reaching significance [H(2) = 5.72, *p* = 0.057] with a mean rank of 40.32 for unaccusatives, 33.66 for unergatives and 26.52 for transitives. Therefore, the argument structure of the verb in use in the sentence seems to have an influence on whether HS speakers adopt the VS answering strategy.

**Table 4:** Production of VS answers by HS participants in sentences with transitive, unergative, unaccusative verbs (in percentages, SD, median).

HS speakers	Transitive	Unergative	Unaccusative
VS mean	36.82	49.77	60.23
SD	32.7	30.37	33.13
Median	34.1	52.8	70.85

Based on the lowest number of VS answers reported alongside transitive verbs, we deduce that the presence of two arguments in the sentence might represent a relevant factor in determining the adopted structures. For this reason, we run a third round of analysis on answers with transitive verbs and observe how objects are realized. Our analysis takes into consideration two factors: (a) the object form, i.e., whether it is produced as a clitic or as a full-fledged lexical DP, and (b) its position with respect to the subject and the verb. As a result, different possible structures are attested for VS answers as well as for SV answers.

Starting with the first factor, i.e., the object form, data reveal that participants from both groups produce objects both as clitics and as lexical DPs (see Table 5).

**Table 5:** Object production analysis (N° and in percentages).

	DP	Clitic
AL2S	192/343 (56.0%)	151/343 (44.0%)
HS	162/320 (50.6%)	158/320 (49.4%)

This observation is particularly interesting in consideration of the fact that lexical DPs were not expected in this context, yet they characterize half of the answers. In the syntactic and pragmatic context offered by the experimental conditions in combination with the videos, objects of transitive verbs always appear in the eliciting questions and are therefore Topics, which express given information in the answer. The repetition of the object Topic as a full lexical noun phrase in the answer is not felicitous; this is characteristically the condition in which the usage of a clitic pronoun is required, as indeed the behavior of native speakers from previous studies confirms (Leonini and Belletti, 2004; Belletti and Rizzi, 2017).

As for the second factor, i.e., the position of the object within the sentence structure, the analysis cannot be carried out without taking into account the subject position and therefore the overall answering strategy in use. In what follows we focus on VS and SV answers in turn and analyze the attested word order in the two strategies^14^. In VS answers the following word orders are attested: Clitic–Verb–Subject (clVS in 19), Object–Verb–Subject (OVS in 20), Verb–Object–Subject (VOS in 21), and Verb–Subject answers with object omission (VS in 22):

(19) L’ ha aperta la ragazza
    obj.CL AUX open.PP the girl

(20) La finestra ha aperto la ragazza
    the window AUX open.PP the girl

(21) Ha aperto la finestra la ragazza
    AUX open.PP the window the girl

(22) Ha aperto la ragazza
    AUX open.PP the girl

As shown in Table 6, speakers from both experimental groups mainly produce answers of the clVS type, thus realizing the object in the appropriate form of a clitic pronoun in VS answers.

**Table 6:** Word orders in VS answers (distribution in percentages).

	clVS	OVS	VOS	Omission
AL2S	95.5	0.0	3.2	1.3
HS	87.7	9.6	2.7	0.0

*clVS, Clitic–Verb–Subject; OVS, Object–Verb–Subject; VOS, Verb–Object–Subject; omission, no object production.*

The following word orders were found in SV answers: Subject–Verb–Object (SVO in 23), Subject–Clitic–Verb (SclV in 24), and Subject–Verb answers with object omission (SV in 25):

(23) La ragazza ha aperto la finestra
    the girl AUX open.PP the window

(24) La ragazza l’ ha aperta
    the girl obj.CL AUX open.PP

(25) La ragazza ha aperto
    the girl AUX open.PP

Table 7 reports which word orders are attested in the SV answers of AL2S and of HS. When SV is the adopted strategy, the object is mainly produced as a full-fledged lexical DP in the postverbal position by both groups. We will comment on this alternative strategy in the discussion section.

**Table 7:** Word orders in SV answers (distribution in percentages).

	SVO	SclV	Omission
AL2S	89.3	7.8	2.9
HS	85.3	13.1	1.6

*SVO, Subject–Verb–Object; SclV, Subject–Clitic–Verb; omission, no object production.*

Summing up, we can conclude that there is a strong relation between the object form and the adopted answering strategy. Objects are consistently produced as clitic pronouns in VS answers and as lexical DPs in SV answers.

## Discussion

The first result in the collected data is that AL2S and HS do not perform as MonoL1 in their production of NISs. Whereas the latter are very consistent in producing VS structures across all verb types, thus setting a clear benchmark for Italian, multilingual speakers typically access a wider range of options. All AL2S and HS participants produce VS answers, although in different amounts, which overall do not reach the rate of MonoL1 speakers. Among the attested alternative options, the most frequent output is the SV one, with a focalized subject in the preverbal position, namely the one described as prototypical in German answers. Although the distribution of VS and SV answers varies across the two groups also in relation to the kind of verb in use, we can clearly see that the two strategies, i.e., postverbal subject and prosodic prominence, are competing in multilingual speakers. The SV constituent order is certainly grammatical in Italian (which is an SVO language as witnessed by the word order in discourse neutral sentences) and multilingual speakers seem to overextend its use also to contexts with New Information Focus subjects. We surmise that this overextension takes place under the pressure of German.

Although we do not know which answering strategies multilingual speakers would produce in German in the very same conditions (no German version of the test is available), we can still assume that overextension works only in one direction. We do not expect to find VS answers of the Italian kind in their German, because the structure would be simply ungrammatical in this language^15^. In contrast, the possibility to use the German SV answers with NISs (respectively, 26.03% for AL2S, and 44.75% for HS on average) is left open (and probably even favored) by the grammaticality of the Subject–Verb word order in Italian. Based on these results, we must conclude that the direction of influence is independent of the status of the language (e.g., German as L2 for AL2S or as the majority language for HS), and it rather depends on the characteristics of the languages involved.

We can now answer the first experimental question regarding the comparison among the three groups and conclude that despite the fact that they are all native speakers of Italian, this factor is not sufficient in assuring the production of the same answering strategy in the relevant discourse contexts. The very consistent behavior of MonoL1 speakers is not replicated by the two multilingual groups, in which we rather found optionality. As for HS, we can assume that optionality is determined by the presence of both languages in their linguistic environment since childhood; whereas we claim that optionality emerges in AL2S as an effect of their advanced acquisition of German as L2. The phenomenon therefore qualifies as a form of attrition in AL2S, which manifests itself as an altered system of coding discourse information into the sentence syntactic structure. In sum, we conclude that being a native speaker of the target language is not sufficient *per se* in shaping answering strategies; in contrast, being a multilingual speaker of languages characterized by different strategies crucially leads to access to different strategies and optionality. Nevertheless, this optionality respects the grammatical constraints of the target language.

Moreover, the results of the competition between the two main alternative strategies, i.e., VS and SV, seem to depend on the argument structure of the verbs in use, as shown by the comparison between AL2S and HS in the four syntactic conditions.

The first and most straightforward observation is that there is actually no competition between alternative strategies in existential structures of the *c’è*/*ci sono* kind (‘there is/there are’). The consistent use of the structure does not leave open any possibility for the emergence of non-target-like structures in this condition, and the subject is realized in the postverbal position, mainly as an indefinite DP. Since existential structures with a postverbal subject are unproblematic even in intermediate speakers of L2 Italian, [as reported by Belletti and Leonini (2004) for their German- and French-speaking learners], it would have been very surprising if our participants had produced alternative strategies in the corresponding condition; and indeed this was not the case.

The picture is more articulated in the three remaining syntactic conditions, namely those with transitive, unergative, and unaccusative verbs: although AL2S and HS essentially adopt the same answering strategies, their distribution varies in the two groups according to different patterns. In particular, AL2S and HS show a significant difference in their distribution of SV answers with transitive and unergative verbs, while the discrepancy between the two groups decreases with unaccusative verbs.

The asymmetric distribution of SV answers between AL2S and HS shows that there actually is a persisting difference between the two groups (Mann–Whitney tests revealed differences between AL2S and HS in the production of SV answers, in particular with transitive and unergative verbs, see the “Results” section for details), such that growing-up as multilinguals and having access to both strategies in the input since childhood plays a role in determining a higher activation of the SV strategy in HS (44.75% across conditions) in comparison to AL2S (26.03% across conditions), and a less frequent activation of the VS strategy (overall at 50.52% in HS, against 61.59% in AL2S). As for AL2S, although VS is still their most active strategy across conditions, they do not behave like monolinguals anymore because multilingualism has enlarged their range of answering strategies.

With respect to the relation between answering strategy and verb type, the pattern becomes clearer when we look at the overall distribution of VS structures: as for AL2S speakers, we see that VS answers are always the preferred strategy from the quantitative point of view, across all verb types; whereas for HS, the number of VS answers increases across the different verb categories. In HS VS answers are at their lowest on transitive verbs (36.82%), they increase with unergative verbs (49.77%), and they are even higher with unaccusative verbs (60.23%); this pattern allows for at least two observations. First, when comparing AL2S and HS the reduced difference between the two groups in sentences with unaccusative verbs is due to a specific increase of HSs’ postverbal subjects with respect to the data attested in the other verb categories. Second, both groups produce the lowest number of VS answers with transitive verbs, thus signaling that the presence of a second argument in the structures might have an impact on the subject position too. We analyze both issues in turns.

As for the former, at the core of the unaccusative hypothesis (Perlmutter, 1978; Burzio, 1986; Belletti, 1988; see also Belletti and Bianchi, 2016 for a more recent overview) is the fact that subjects of unaccusative verbs present different properties than the subjects of transitive and unergative verbs; specifically, they are first merged as the internal argument rather than as the external one; moreover, (indefinite) subjects of unaccusatives can be licensed internally to the verb phrase, thus remaining in the postverbal position. Based on the collected data, we assume that the specific property of the verb argument structure favors the production of postverbal subjects in HS. In other words, we suggest that the production of VS structures by HS might be determined, at least in part, by the property of unaccusative subjects *per se*^16^.

As for the second issue concerning transitive verbs, the presence of a second argument in their structure seems to increase optionality; this leads us to the discussion of the fourth research question we raised, namely the one concerning the realization of the object in sentences containing NISs. Under the discourse conditions of the experimental task, the object of transitive verbs is present in the questions together with the verb, thus qualifying as a given Topic in the answer. In the elicited pragmatic condition, Topic-like objects are usually realized as clitic pronouns in Italian. For instance, Leonini and Belletti (2004), elicited answers to questions characterized by topic-like objects from monolingual Italian speakers and observed that participants were very consistent in producing clitic objects in their outputs (91%), and only rarely reproduced the complete lexical DP (7.7%) they had been exposed to in the question. However, in the present study participants’ answers only partially meet this expectation: both AL2S and HS produce objects either as clitic pronouns or as full-fledged lexical DPs in comparable amounts. What is most striking in the data is that both groups behave very much alike in their object realization because they all show a strict correspondence between object form and subject position: in VS answers, objects are mainly produced in the form of clitics; while in SV structures, they are produced as lexical DPs in the postverbal position, thus resulting in the SVO word order^17^. Among the alternative solutions, we would like to briefly comment on the fact that HS also produce the OVS word order with lexical objects (9.6% of VS answers), thus realizing a pattern that we interpret as possibly reflecting a V2-type structure in their heritage language. The same strategy is not attested in the AL2S group, thus indicating that attrition has not yet determined the onset of the V2 computation in Italian. If we now consider results with transitives alone, it would be hard to tell whether the object form (clitic/lexical DP) determines the subject position (postverbal/preverbal) or the other way around. However, due to the fact that the production of VS is not at ceiling in the unergative and unaccusative conditions, presence of the object cannot anyway be the only cause for the production of preverbal subjects.

In light of the above discussion with respect to the performance of HS, we would like to claim that the performance on unergative verbs should be taken as the benchmark for the production of postverbal subjects in HSs. Transitive structures reduce the number of postverbal subjects, most likely as a reflex of the presence of the object, mostly when it is realized as a lexical DP and not as a clitic, as its discourse status would require. In contrast, the number of VS answers increases with unaccusative verbs because of the possibly wider source for postverbal subjects (as either internal to verb phrase in the merge position as internal argument of the verb, or as a NIS in the specfier of the low New Information Focus position).

Finally, we would like to point out another aspect of the overall performance of HS participants. Although their production of VS answers is relatively limited, the range of answering strategies they produce is the same as in AL2S. Therefore, there is nothing impoverished in their performance. The observation is relevant because it further supports the claim put forth by Kupisch and Rothman (2016; see also Rinke and Flores, 2014; a.o.) against the description of HS as speakers characterized by an incomplete grammar^18^. As far as the production of answering strategies is concerned, nothing seems to be incomplete or incorrect in their outputs, and the asymmetry must therefore be explained as a consequence of their multilingual grammar(s). The claim that HS speakers’ grammar is not incomplete is based on the comparison with AL2S, who certainly achieved a complete maturation of their native language while growing-up as monolinguals. Since we do not talk about incomplete grammar for AL2S, we do not do so for HS either. However, we could have reached this conclusion, i.e., that HS have an incomplete grammar, if we had only compared their performance to that of MonoL1 speakers: this shows the importance of choosing a richer array of speakers to which HS should be compared. Note that AL2S represent the prototypical source of input for HS in the familial environment; under this perspective, the similarity between AL2S and HS can be further evaluated as the sign of a rather successful acquisition of the target language by HS based on the quality of the input they received. The mild differences between AL2Ss and HS rather relate to the multilingual setting of the HS linguistic development.

## Conclusion

As to the variables, we considered and which are factors contributing to language dominance, we can conclude that nativeness, multilingualism and amount of input in L1 acquisition do not play equivalent roles when seen through the lenses of answering strategies.

In the present study, we showed that AL2S and HS have access to a wider range of answering strategies with respect to monolingual speakers, thus indicating that nativeness does not guarantee the production of homogeneous answering strategies (as attested in monolingual speakers). This leads to the second variable we considered, i.e., multilingualism: we interpret the presence of SV answers in AL2S as a consequence of the multilingual competence they developed in their adult life; the activation of the German SV word order while computing NISs in Italian shows that alternative strategies can take over the VS strategy in place from childhood (Belletti, 2007), thus resulting in attrited performance in L1. Based on these results, we conclude that language dominance within specific linguistic phenomena can possibly be undermined throughout the lifespan under the pressure of advanced L2 acquisition.

The role of the amount of input in L1 acquisition emerges from the data on the use of alternative strategies: since the two groups differ in the distribution of SV answering strategies, we claim that the condition in which L1 Italian is acquired (as the only L1 for AL2S or as the heritage language for HS) plays a role in determining how active the SV strategy is in Italian, and therefore also how (un)stable the production of postverbal NISs is in multilingual speakers. In particular, differences between AL2S and HS in the production of answering strategies can be better explained under an analysis that takes into consideration the argument structures of the verbs in use, with unaccusative verbs particularly favoring the production of postverbal subjects in HS.

Overall, optionality between VS and SV is attested in all multilingual speakers. Based on these results, we claim that the competence of HS is target-like because AL2S represent their typical source of input during early language development; therefore, nothing is missing, incorrect or incomplete in the grammar of HS.

Finally, the analysis of answers with transitive verbs showed a relation in both groups between the subject position and the object form: AL2S and HS consistently produced object clitic pronouns in combination with postverbal subjects, and lexical object DPs in combination with preverbal subjects. Again, the performance of multilingual speakers differed from what is expected in monolingual speakers (i.e., Topic-like objects realized as clitic pronouns), thus suggesting that this further discourse-related structure is an unstable domain in multilingualism. The phenomenon is well- known from L2 acquisition and should be further explored in future research in the domain of L1 attrition and heritage language, with a particular attention to its interaction with other arguments in the sentence. This is the topic of current research.

## Ethics Statement

Ethics approval for this research was not required as per the guidelines of the Faculty of Modern Languages at Goethe University as well as per national regulations. The test did not involve any risk on participants’ physical, mental and social integrity. All adult subjects gave written informed consent to participate in the study and could withdraw consent to use the collected data at any time without reprisal.

## Author Contributions

AB conceived the idea of the study and wrote the Sections “Answering Strategies” and “Subjects at the Interfaces: Previous Results From Multilingual Speakers.” IC collected the data and wrote the Sections “Research Questions,” “Materials and Methods,” “Results,” and “Discussion.” CP wrote the Sections “Introduction” and “Conclusion.”

## Conflict of Interest Statement

The authors declare that the research was conducted in the absence of any commercial or financial relationships that could be construed as a potential conflict of interest.

## Footnotes

1 Or V- to the relevant non-finite past participial morphology as in examples like (2) containing a periphrastic Aux + Pst Prt tense. The head position targeted by the past participle is still higher than the discourse related vP-periphery.

2 The availability of so called ‘subject inversion’ yielding the VS order and the null subject status of the language is indeed a core correlation in the classical literature on the null subject parameter (Rizzi, 1982; Jaeggli and Safir, 1989). The null subject property of the language is a necessary condition to allow for a VS order such as the one found in Italian type answering strategy. Notice, however, that nothing in principle rules out the grammatical possibility for a null subject language to also allow or even prefer a SV-type answering strategy in similar contexts. Bulgarian may precisely be a case in point according to the recent discussion in Genevska-Hanke (2017).

3 As discussed in the references quoted, many other diverse languages adopt the cleft strategy, among which, e.g., Norwegian, Malayalam, Japanese, Brazilian Portuguese.

4 See Belletti (2007) for the conclusion that the different answering strategies are in place from very early ages, based on a search on CHILDES (MacWhinney, 2000).

5 Rinke and Flores (2018) report further cross-linguistic data on this issue: the authors claim that in European Portuguese the acquisition of correct interpretation of subjects takes longer for overt pronouns than for null pronouns both in monolingual and in (German–Portuguese) bilingual children.

6 The phenomenon is even more striking when data analysis takes into consideration the argument structure of the verbs in use: cleft production by L1 speakers of French is particularly high in elicited Italian answers containing either a transitive verb (88%) or an intransitive verb of the unergative kind (80%). Differences among verb types will be further addressed in Section “Results.”

7 These data are confirmed also by an independent study run with L1 speakers of Polish: Labuz (2012) ran the Polish version of the test in use in Belletti and Leonini (2004) with 16 monolingual L1 speakers of Polish and found that they produced VS structures in 84% of the answers with NISs. The strategy remains active in Polish speakers of L2 Italian (15 participants), who perform almost target-like in Italian (95% of VS answers).

8 Except for sentences that include existential structures of the *c’è*/*ci sono* kind (i.e., ‘there is/there are’), as was also the case for the non-advanced French and German groups discussed earlier.

9 Although overproduction of overt pronominal subjects can also be detected in their oral production (Belletti et al., 2007, p. 672).

10 In 10 families both parents were native speakers of Italian; in 10 families one parent was a native speaker of Italian and one parent was a native speaker of German; in 2 families one parent was a native speaker of Italian and the other parent was a native speaker of a further minority language (i.e., Hungarian or Polish), but this was not transmitted to the child.

11 For reasons of space we cannot discuss here how the amount of input in the target languages can be measured in a bilingual context, but see Grüter and Paradis (2014) for detailed discussions.

12 One participants also reported to have lived for 1 year in an English-speaking country during childhood.

13 The two texts in use differ for the two languages (one was not the translation of the other), such that the comprehension of one text could not bias the completion of the other one.

14 The reasons for excluding answers of the OTHER kind from the analysis is rather straightforward: as reported above, this category mainly includes passives and reduced cleft. In passive structures, the internal argument is promoted to the subject position and is most likely produced as a fully fledged lexical DP, while in reduced cleft of the kind produced by our participants (see example 16 above) the object is just not there.

15 In German expletive *pro* and low lexical subjects are limited to impersonal passive structures (Hubert, 1989), which are not relevant in the pragmatic conditions elicited in the present study.

16 Given the indefiniteness requirement holding on the internal argument of unaccusatives, VS answers with an indefinite subject could have the subject in the VP-internal argument position of its first merge.

17 The VOS order, only marginally possible in Italian, is attested very sporadically, in the HS group only. The VSO order, impossible in Italian for principled reasons (Belletti, 2004), is totally absent in both groups. This is an interesting convergence with the results in the original study on L2 Italian by Belletti and Leonini (2004).

18 See also Di Venanzio et al. (2016) and Schmitz et al. (2016), who argue against incomplete acquisition of the heritage language in Italian–German HS who grew up in Germany. Their claim builds on the lack of significant differences with respect to monolinguals when the production of lexical/pronominal objects and that of null/overt subjects are analyzed in their spontaneous speech.
